# Weakly Cross-Linked Anionic Copolymers: Kinetics of Swelling and Water-Retaining Properties of Hydrogels

**DOI:** 10.3390/polym15153244

**Published:** 2023-07-30

**Authors:** Leonid Iliasov, Andrey Shibaev, Irina Panova, Petr Kushchev, Olga Philippova, Alexander Yaroslavov

**Affiliations:** 1 Department of Chemistry, M.V. Lomonosov Moscow State University, Leninskie Gory 1, 119991 Moscow, Russia; igpan@mail.ru (I.P.); yaroslav@belozersky.msu.ru (A.Y.); 2 Department of Physics, M.V. Lomonosov Moscow State University, Leninskie Gory 1, 119991 Moscow, Russia; shibaev@polly.phys.msu.ru (A.S.); phil@polly.phys.msu.ru (O.P.); 3 Department of Chemistry, Karaganda E.A. Buketov University, University Street 28, 100028 Karaganda, Kazakhstan; 4 Department of Chemistry, Voronezh State University, Universitetskaya Sq. 1, 394018 Voronezh, Russia; peter.kuschev@gmail.com

**Keywords:** cross-linked polymers, hydrogels, sand, soil, swelling, water retention, water capacity, rheology

## Abstract

Six cross-linked copolymers consisting of sodium acrylate, N-acrylamide, starch fragments and a cross-linker were synthesized, potentially suitable for use in agriculture as superabsorbents. The copolymers had the same content of carboxyl groups equal to 6.2 mmoles per 1 g of copolymer and the content of cross-linker (Q) varied from 0.04 up to 1 wt.%. The copolymers swelled in a pH 6.5 aqueous buffer solution thus giving hydrogel particles, which were characterized by a set of methods including gravimetry, rheometry, swelling pressure analysis, equilibrium centrifugation and water retention analysis with the following main conclusions. An increase in Q decreases the equilibrium degree of swelling. When swelling in a solid substrate, sand or soil, the equilibrium degree of swelling shows the maximum at Q = 0.14 wt.%. The cross-linking degree controls the swelling pressure of hydrogels and water-retaining properties of solid substrates with embedded hydrogels; in both cases, the maximum effects are observed at Q = 0.14 wt.%. These extreme dependences set the algorithm for synthesis of polymeric superabsorbents and optimization of their operational characteristics.

## 1. Introduction

Superabsorbent hydrogels (SAHs) are among the promising materials for agrochemistry and crop production due to their ability to absorb and retain significant amounts of water [1,2,3,4]. SAHs are soft materials whose properties are determined by the chemically cross-linked polymer network [5,6]. The main practically-oriented functions of SAHs are to reduce the consumption of irrigation water and increase the moisture available for plants in soils [7,8,9]. In agriculture and crop production, synthetic cross-linked copolymers, composed of acrylamide and negatively charged acrylic acid/acrylate units, are widely used [2,3,4]. Acrylamide units are well solvated by water and form hydrogen bonds with water molecules [2,10]; this, together with the high negative charge created by dissociated acrylate units, provides excellent swelling of such copolymers in aqueous solutions [5,6].

Recently, biodegradable ionic polymers (polyelectrolytes, PEs) of natural origin are increasingly considered as water-retaining agents [1,4,11,12,13,14]. However, biopolymers such as cellulose, starch, alginates, and lignin alone are not capable of forming SAHs [1,4]. For this reason, natural polymers are combined with synthetic ones to improve water retention properties. Acrylamide/acrylate fragments improve the mechanical properties of the combined hydrogels in the swollen state [1,4]. Additionally, such copolymers can reduce the environmental load on the soil due to biodegradation of mixed compositions [1,4,11].

The driving force for the polymer network swelling is the gradient of effective osmotic pressure [5,6]. The process develops until the swelling pressure of the gel particles becomes equal to the external pressure [5,6,15]. The superabsorbent capacity of cross-linked polymers is associated not only with the chemical nature of the monomer units, but also with a low content of cross-linking agents (low degree of cross-linking). When added to water, the polymer chains, initially assembled into compact coils, straighten out, which provides an increase in the polymer network volume; the latter decreases with rising the degree of cross-linking [2,5,6,16,17]. However, in agricultural technologies, dry cross-linked copolymers are mixed into the soil, where they are forced to swell in a limited pore space under the pressure of soil particles. Therefore, in recent years, there has been an increased interest in studying the behavior of SAH in the matrix of solid particles, both model and natural [15,18,19,20,21]. It has been shown in particular that the swelling of cross-linked polymers in the limited space and under the external pressure is reduced compared to the free state [4,15,18,22,23,24,25]. The swelling capacity is also reduced as the particle size of the solid substrate decreases [15,18]. These observations indicate a relationship between water-retaining properties and mechanical characteristics of the swollen polymer networks.

In the current article, we describe the synthesis of six weakly cross-linked copolymers with the same content of anionic groups and the content of cross-linking agent (Q) varied from 0.04 to 1 wt.%. We quantify the effect of the cross-linking degree on the kinetic and equilibrium swelling of copolymers (α) in water and in the solid substrates, sand and soil. We show that the α value in sand and soil does not decrease progressively when increasing Q, but passes through the maximum at Q = 0.14 wt.%. We also show that the cross-linking degree controls the swelling pressure of hydrogels and water-retaining properties of solid substrates with embedded hydrogels; in both cases, the maximum effects are observed at Q = 0.14 wt.%. These extreme dependences were not described before. They set the algorithm for the synthesis of polymeric superabsorbents and optimization of their operational characteristics.

## 2. Materials and Methods

Acrylic acid (AA), starch, potassium hydroxide and potassium persulfate (all from Vekton, Saint Petersburg, Russia), acrylamide (AAm) and a cross-linking agent, N,N′-methylene-bis-acrylamide (bisAAm) (both from Acros Organics, Trenton, NJ, USA), KBr and concentrated HCl (both from Reakhim, Moscow, Russia) were used as received.

Cross-linked ACP# copolymers were synthesized using the procedure described earlier [22]. Briefly, two aqueous solutions were prepared separately: Solution 1 through addition of AAm and bisAAm to a solution of AA and KOH in distilled water, Solution 2 through dissolving starch in distilled water followed by addition of an aqueous solution of potassium persulfate. Then, Solution 1 and Solution 2 were combined and the mixture was heated up to 70 °C in order to initiate formation of radical sites along the starch chain with further graft polymerization of co-monomer/cross-linker mixtures. The product (network copolymer) was dried to constant weight, and the quantitative yield was found (within the accuracy of the gravimetric method ±0.1%). The dried product was crushed, and the fraction with a particle size of 0.25–0.5 mm was used. With this procedure, six copolymers were synthesized with the content of cross-linking agent (Q) equal to 0.04 (ACP#1), 0.08 (ACP#2), 0.14 (ACP#3), 0.2 (ACP#4), 0.4 (ACP#5) and 1 wt.% (ACP#6). The composition of ACP#s were confirmed by IR spectroscopy using Specord M 80 spectrometer (Carl Zeiss, Oberkochen, Germany), the content of carboxylic groups in the ACP#s was quantified with potentiometric titration [22] that gave the same value, (6.2 ± 0.1) mmoles per 1 g of ACP#, for all six copolymers that corresponded to the weight content of 27.9%.

Fine-grained monomineralic quartz sand with a grain size of 0.1–0.2 mm (OPT6, Moscow, Russia) was repeatedly washed with bi-distilled water before use. A soil sample was taken in the upper ten-centimeter layer on the experimental field near Erokhino village in the Pskov region (Russia) with section coordinates: 56.129474 North and 31.372435 East. The sample was grinded and fractionated as described earlier [26]. The granulometric composition (particle size distribution) of the soil sample showed 85.7% of sand (a 0.05–1 mm fraction) with a minor content of physical clay (a < 0.002 mm fraction) and dust (a 0.002–0.05 mm fraction).

An equilibrium degree of free (“in water”) ACP# swelling (α) was quantified gravimetrically using a Shimadzu MOC-63U moisture analyzer (Shimadzu, Kyoto, Japan) as described in [22]. The α values were calculated as:

α = (m_sw_ − m)/m, (1)

where m_sw_ and m are the masses of swollen in water and dry ACP#, respectively.

Swelling of ACP# in a limited pore space of sand and soil (hereinafter referred to as “substrate”) and the effect of ACP# on the water-retention properties of sand and soil were examined with a Shimadzu MOC-63U moisture analyzer [22]. A water content in the samples was calculated as:

W = (M_sw(mix)_ − m_s_ − m)/M_(mix)_, (2)

where M_sw(mix)_ and M_(mix)_ are the masses of wet and dry polymer-sand/soil mixture and m_s_ is the mass of dry sand/soil, and compared with the maximum water content in the original sand/soil (W):

W = (m_sw(s)_ − m_(s)_)/m_(s)_, (3)

where m_sw(s)_ and m_(s)_ are the masses of wet and dry sand.

An equilibrium degree of ACP# swelling “in the substrate” (α_lim_) was calculated as:

α_lim_ = (M_sw(mix)_ − m_sw(s)_ − m)/m. (4)

Strain dependences of storage modulus G′ and loss modulus G″ for ACP#s were measured using a Physica MCR 301 rotational rheometer (Anton Paar, Graz, Austria) in a plate–plate cell with the sensor diameter of 25 mm at 26 °C. Shear stress was determined at a fixed frequency ω = 10 s^−1^ and a constant deformation amplitude of 0.005.

**Figure 1:**
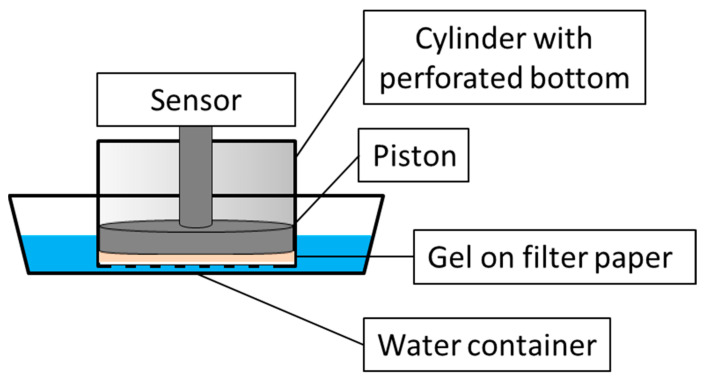
Design of the measuring cell to control the swelling pressure of copolymer granules.

Additionally, a model experiment was carried out to control the swelling pressure of copolymer granules in a limited space with non-deformable walls. For this, a special measuring cell was designed (Figure 1), which included (1) a hollow cylinder with an internal cavity, 56 mm in diameter, and a bottom perforated with 1.4 mm holes to penetrate water and (2) a movable piston with a 56 mm diameter to register the pressure created by the copolymer hydrogel.

The bottom of the cylinder was covered with a filter paper and 1.5 g of the copolymer granules were evenly distributed on its surface. The piston was lowered down until the distance between the copolymer and the piston reached 50 μm. Then, the cylinder was put in a container with a 0.001 M aqueous phosphate buffer with pH 6.5 that induced the penetration of water into the cylinder and the swelling of the copolymer. The resulting hydrogel pushed the piston, a time-dependent pressure being recorded by a sensor connected to an Ametek Lloyd LS5 tensile testing machine (Ametek, Berwyn, PA, USA). Finally, the pressure became so great that the swollen granules broke down and leaked through the holes in the bottom of the cylinder that caused the pressure to fall.

In the water retention experiments, an aqueous copolymer formulation was added to a sand/soil sample, then thoroughly mixed and dried to constant weight [22]. Water retention curves for polymer-treated and original sand/soil were obtained by equilibrium centrifugation [27]. Approximation of the curves was conducted using the van Genuchten model, which describes their S-shape forms with one inflection using a minimum number of approximation parameters [28].

All experiments were repeated 3–5 times. Statistical data processing with Microsoft Excel program included the calculation of mean values and standard deviations.

## 3. Results and Discussion

### 3.1. Swelling of Cross-Linked Anionic Copolymers in Aqueous-Salt Solutions

**Figure 2:**
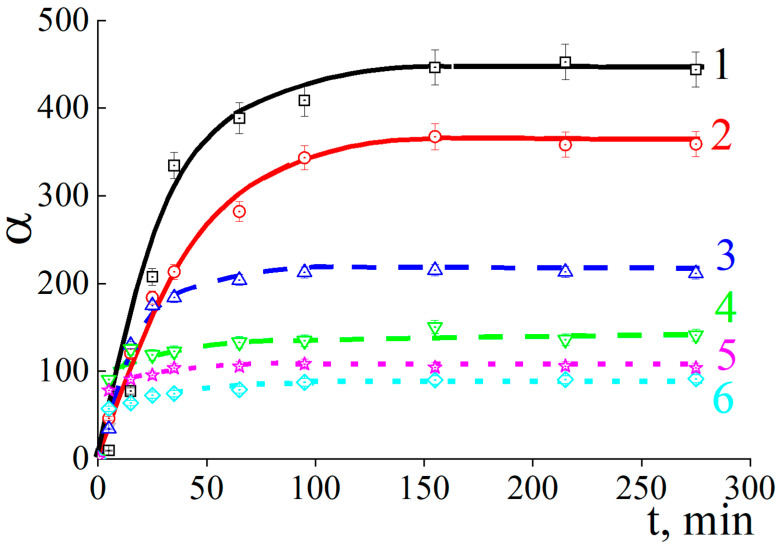
Time-dependent degree of swelling for cross-linked copolymer granules in 10^−3^ M phosphate buffer with pH = 6.5. ACP#1 (1), ACP#2 (2), ACP#3 (3), ACP#4 (4), ACP#5 (5) and ACP#6 (6).

We started with the kinetics of ACP# swelling in an aqueous buffer solution. For all studied ACP#s, the degree of swelling increased with time and eventually reached the ultimate values (Figure 2). As expected, the equilibrium degree of swelling α progressively decreased with increasing content of cross-linking agent Q. At the same time, the higher the Q value, the faster the ultimate α value was achieved: 150 min for ACP#1 and 20 min for ACP#6.

**Figure 3:**
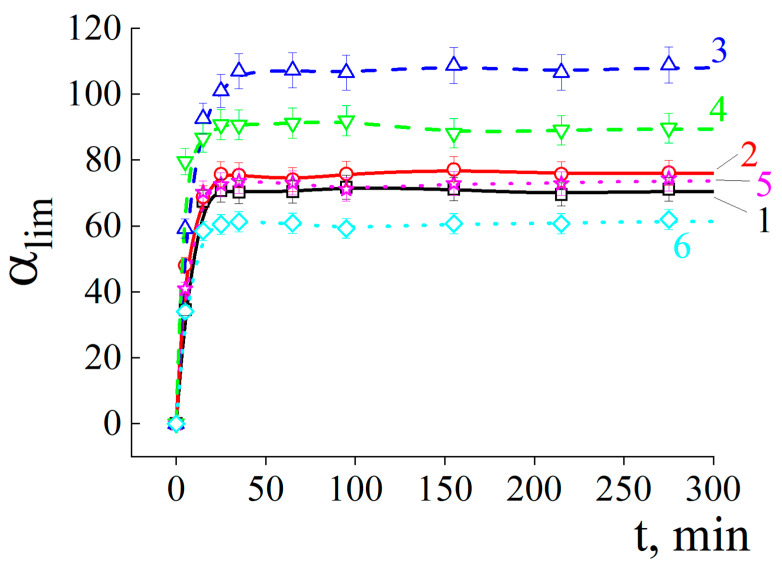
Time-dependent degree of swelling for cross-linked copolymer granules mixed with sand in 10^−3^ M phosphate buffer with pH = 6.5. Sand/copolymer weight-to-weight ratio of 100. ACP#1 (1), ACP#2 (2), ACP#3 (3), ACP#4 (4), ACP#5 (5) and ACP#6 (6).

After that, dry copolymers were mixed with the sand so that the copolymer/sand weight-to-weight ratio was the same for all samples. Each copolymer/sand mixture was put in a glass cylinder with an open (perforated) bottom, through which water penetrated the cylinder. This caused the copolymer to swell and occupy the maximum volume inside the pore space. The kinetics of ACP# swelling in the substrate was described by the curves (Figure 3) with the profiles close to those for the ACP# swelling in water (cf. with the curves from Figure 2). However, the equilibrium degree of ACP# swelling in the sand initially increased, and reached the maximum for ACP#3 (Figure 3), and then decreased for ACP#s with higher Q contents, while the time to reach the equilibrium was noticeably reduced.

**Figure 4:**
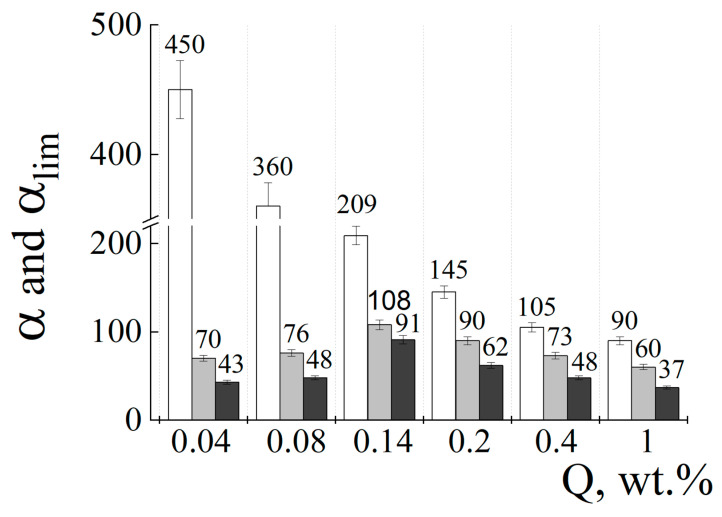
Degree of ACP# copolymer swelling in 10^−3^ M phosphate buffer with pH = 6.5 vs. Q. Free swelling in the absence of sand/soil (white columns), swelling in the confined space in sand (gray columns) and soil (black columns). Substrate/ACP# weight-to-weight ratio of 100.

Similar kinetic experiments were carried out with another substrate, soil. The α values for six ACP#s swollen in water and the two substrates, sand and soil, are summarized in Figure 4. Three things draw attention to themselves. First, the swelling degree for the copolymers in the sand (gray columns) and soil (black columns) decreased compared to this parameter for the copolymers swollen in water (white columns). For example, the α values dropped from 450 in water down to 70 in sand and 43 in soil for ACP#1 (Q = 0.04 wt.%), and from 90 in water down to 60 in sand and 37 in soil for ACP#6 (Q = 1 wt.%). Second, a progressive decrease in α with increasing Q was detected for the copolymers swollen in water but “bell-shaped” histograms with the maximum for ACP#3 with Q = 0.14 wt.% for the copolymers swollen in the sand and soil. Third, the figure showed higher α values for the copolymers swollen in sand in comparison with the copolymers swollen in soil.

The swelling of the anionic cross-linked copolymers in water is determined by a degree of cross-linking: the higher Q, the lower α [6]. The situation changes, when the copolymers are mixed with the sand. Now they are forced to swell in a limited space—in the pores between the sand particles. Under these conditions, the swelling is determined by a balance between the mechanical strength of the swollen polymer and the resistance from the sand particles. The slightly cross-linked ACP#1 and ACP#2 give highly swollen but mechanically fragile hydrogels, which cannot effectively compete with durable sand particles. This results in a sharp decrease in α for both ACP#/sand mixtures (Figure 4). Stronger ACP#s hydrogels, from ACP#3 to ACP#6, are capable of occupying more interpore space, although the swelling appears to be less than for the same copolymers in water. Eventually, the interplay of the polymer strength and resistance from sand results in a weak “α_lim_ vs. Q” plot with the maximum at Q = 0.14 wt.% (gray columns in Figure 4).

A similar mechanism is true for the copolymer-soil systems (black columns in Figure 4). A catastrophic drop in the α value for the ACP#1 and ACP#2 in the soil is observed while the α values for other copolymers, from ACP#3 to ACP#6, do not shrink as much. These changes finally lead to the bell-shaped profile of the “α_lim_ vs. Q” plot as in the case of the copolymer-sand systems.

The only difference between the behaviors of copolymers in the sand and soil manifests itself in the absolute values of α for these two substrates. The α values are always higher for the sand than for the soil. Apparently, it is due to more free pore space in the sand samples, constructed from particles whose size exceeds the size of soil particles as shown in our previous work [22,27].

**Figure 5:**
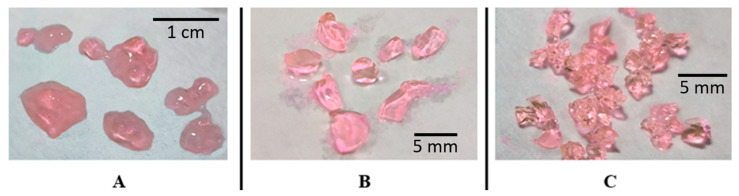
Photos of copolymer particles swollen in an aqueous solution with dissolved Rhodamine 6 g dye. ACP#1 (**A**), ACP#2 (**B**) and ACP#6 (**C**) hydrogel.

Different mechanical properties of the hydrogels could be observed even visually. The weak ACP#1 hydrogel with the smallest Q spread over the surface and formed droplets (Figure 5A). The ACP#2 hydrogel retained its shape on a hard surface (Figure 5B). Even more cross-linker resulted in elastic hydrogel granules (Figure 5C).

**Figure 6:**
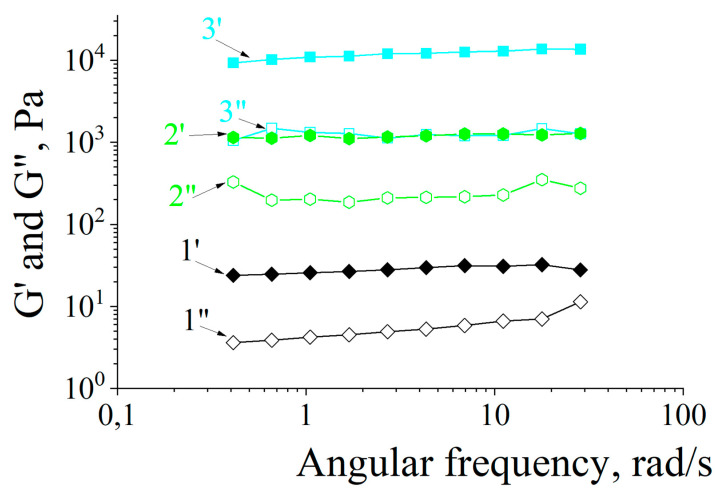
Storage modulus G′ (filled symbols) and loss modulus G″ (white symbols) vs. angular frequency of shear oscillations for ACP#1 (1′ and 1″), ACP#4 (2′ and 2″) и ACP#6 (3′ and 3″) hydrogels.

Rheological properties of the hydrogels were examined via analyzing strain dependences of storage modulus G′ and loss modulus G″ on the angular frequency of shear oscillations. As an example, in Figure 6 the experimental data for three samples are shown: ACP#1, ACP#4 и ACP#6. For each ACP#, the frequency dependencies of the G′ and G″ values are parallel, and the G′ values are always over the G″ values. These results indicate the viscoelastic behavior of all studied hydrogels with different Q values.

**Figure 7:**
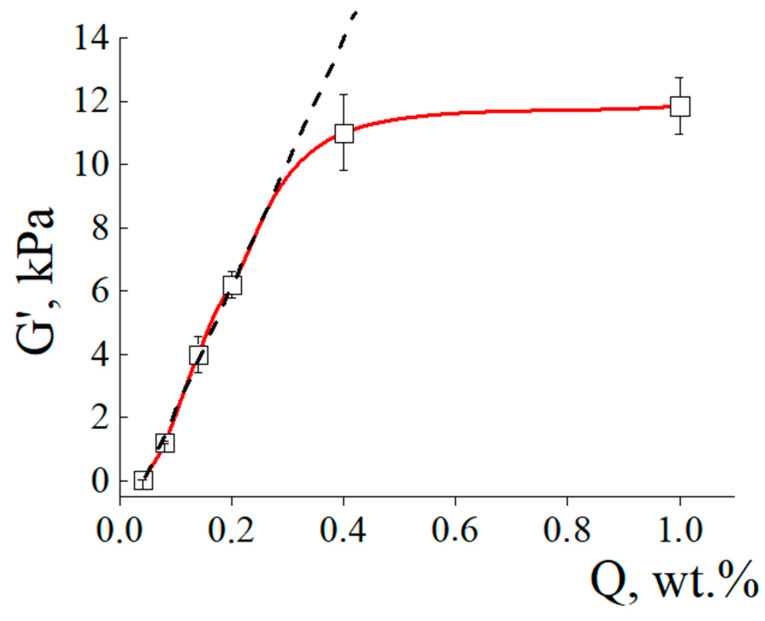
Storage modulus G′ vs. content of cross-linking agent Q for ACP# hydrogels.

The storage modulus G′ characterizes the elastic mechanical energy of the sample. The total G′ vs. Q plot (Figure 7) showed a linearity up to Q = 0.3 wt.% and then only slightly increased. A similar result was described earlier [29]. This was assumed to be due to a large number of inhomogeneities in the polymer networks with a high content of the cross-linking agent.

The storage modulus for the most slightly cross-linked ACP#1 was only of 25 Pa. For ACP#6, it was equal to 11.8 kPa, which was nearly three orders of magnitude higher than the value for ACP#1. Recall that it is the mechanical stability of the hydrogels that determines their ability to withstand the external pressure of the solid substrate. For this reason, the hydrogels with the lower content of cross-linking agent Q swell weakly in the sand and soil that finally resulted in the bell-shaped α_lim_ vs. Q plot as shown in Figure 4.

**Figure 8:**
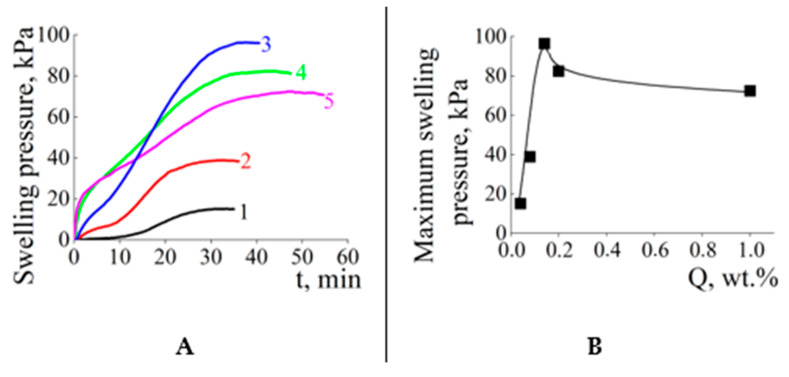
(**A**) Time-dependent swelling pressure of ACP# copolymer granules placed in the metal cylinder. ACP#1 (1), ACP#2 (2), ACP#3 (3), ACP#4 (4) and ACP#6 (5). 10^−3^ M phosphate buffer with pH = 6.5. (**B**) Maximum swelling pressure vs. content of cross-linking agent Q.

In order to control the pressure from the solid substrate, the following model experiment was carried out. The granules of dry copolymers were placed in a rigid metal cylinder cell with small holes in the bottom. Water penetrated through the holes into the cell, that led to the swelling of the copolymer and the appearance of additional force, which increased the pressure on the top cover of the cell. Here, the walls of the metal cylinder replace the sand and soil particle surface in the above-described experiments on the swelling of copolymers pre-mixed with both substrates. The “pressure vs. time” plots for the copolymers swollen in the metal cylinder are shown in Figure 8A.

In all cases, the swelling of copolymers was accompanied by raising the pressure; however, the profiles of the “pressure vs. time” plots were different. For the ACP#1, the pressure became measurable only 10 min after water addition (curve 1), and 30 min required to reach the maximum pressure. In contrast to this, other ACP# samples began increasing the pressure just after water addition (curves 2–5) with the maximum pressure after 30–45 min. Such behavior is obviously associated with different Q values, which affects their ability to absorb water and form mechanically strong hydrogels. The slightly cross-linked ACP#1 requires more water and more time to straighten the polymer chains and produce a hydrogel capable of pressing down on the top cover. Other copolymers, with denser cross-linking and shorter polymer chains, form rather strong hydrogels, which begin pressing the top cover just after water addition.

As to the maximum pressure on the top cover (P_max_), it showed an extremum at Q = 0.14 wt.% (Figure 8B). This observation is in agreement with the above “bell-shaped” plot describing a relationship between the swelling of copolymers mixed with sand/soil and content of the cross-linking agent in copolymer Q (gray and black columns in Figure 4).

### 3.2. Water-Retaining Properties of Anionic Copolymer Hydrogels

The swelling of the cross-linked copolymers determines their water-retaining properties in various soil substrates. The latter are important in particular when discussing the ability of hydrogels to accumulate water needed for germination and developments of plants. Water retention capacity of sand and soil was represented conventionally as a correlation between water content in the sand (W) and a logarithm of pressure (pF) specified by sample centrifugation as described elsewhere [27]. The higher the W value at the same pF, the higher the ability of the soil to accumulate and retain water.

**Figure 9:**
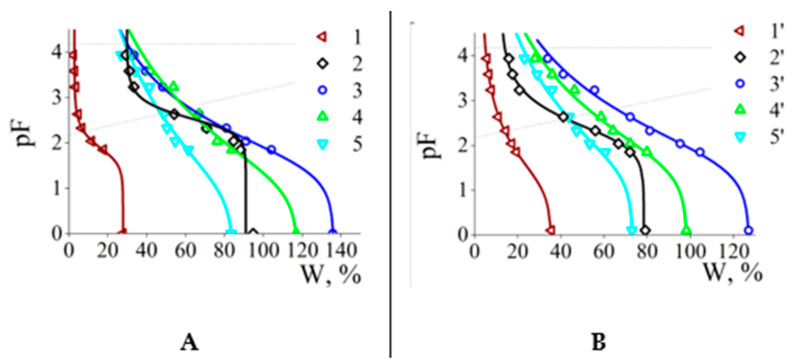
Water retention curves for sand (**A**) and soil (**B**) treated by ACP#. No ACP# (1 and 1′), ACP#1 (2 and 2′), ACP#3 (3 and 3′), ACP#4 (4 and 4′) and ACP#6 (5 and 5′). The samples were saturated with 10^−3^ M phosphate buffer, pH 6.5. Dotted lines show the Voronin cross-sections [30] for calculating soil-hydrological constants (FWC and WP, in the sequence from bottom to top). Substrate/ACP# weight-to-weight ratio 100.

Experimentally found W values were approximated by S-shaped water retention curves (WRCs) according to the Van Genuchten model [28]. In Figure 9A the results are shown for the sand alone (control) and four sand-ACP# systems. The maximum water capacity of the initial sand at zero external pressure was of W_max_ = 27% (Figure 9A, curve 1). An increase in pF was accompanied by decreasing the water capacity value, which became negligible at pF = 2.5 corresponded to a pressure of ~31 kPa.

Addition of ACP#1 with Q = 0.04 wt.% to the sand shifted the WRC to the right, so that W_max_ = 95% (Figure 9A, curve 2). The ACP#3 (Q = 0.14 wt.%) moved the WRC to W_max_ = 136% (Figure 9A, curve 3), whereas the ACP#4 (Q = 0.2 wt.%) and ACP#6 (Q = 1 wt.%) decreased W_max_ down to 116% (Figure 9A, curve 4) and 83% (Figure 9A, curve 5). As a whole, the profile of a “W_max_ vs. Q” plot with the maximum at Q = 0.14 wt.% expectedly resembled the profile for the “the “α vs. Q” plot from Figure 4 (gray columns). A rise in pF decreased W, which took the minimum value of 30–40% at pF = 4.18. The addition of copolymers to the soil led to similar changes in water retention curves (Figure 9B) with a bell-shaped “W_max_ vs. Q” plot with the maximum at Q = 0.14 wt.% thus resembling the “α vs. Q” plot profile from Figure 4 (black columns).

The data of Figure 9 allowed for the estimation of important hydrophysical indicators of the copolymer/sand and copolymer/soil mixtures. For this, two secant lines were drawn in Figure 9 according to the equations: pF = 2.17 + W/100 (1) and pF = 4.18 (2). The intersection of the first with WRC gives the lowest (equilibrated) moisture capacity for the sample (field water capacity, FWC), the intersection of the second gives the moisture inaccessible to plants (wilting point, WP) [30]. The difference between these values determines the range of moisture available to plants (available water range) AWR = FWC − WP.

In Table 1 are summarized the results of WRC analysis for the sand and soil and both solid substrates mixed with six polymer hydrogels. In the table, the maximum water capacities are shown for the sand and soil at zero external pressure, W_max_. In the initial sand, the W_max_ was equal to 27%; however, the FWC did not exceed 4%, and the WP was only of 1%. As a result, the AWR = FWC − WP = 3 wt.% that reflected a low water retention capacity, typical for unstructured sandy substrates [31]. The corresponding parameters for the initial soil are given below: W_max_ = 36%, FWC = 4%, WP = 5 and AWR = 9 wt.%. The latter indicated a bit higher low water retention capacity of the soil in comparison with the sand.

The hydrogels raised all hydrophysical parameters upward and, most importantly, increased the AWR values responsible for plant germination and development. For aggregated fertile loamy soils, the AWR value has been reported to be of 16% [31]. We noted that the use of hydrogels allowed for exceeding this indicator up to 18–31% for the sand and 22–33% for the soil (Table 1).

Notably, all hydrophysical indicators, W_max_, FWC, WP and AWR, reached their maximum at Q = 0.14 wt.%. Larger and smaller values of Q reduced the indicators by 10–50%. These changes reflect an ability of hydrogels to absorb and retain water in the mixture with sand and soil and correlate well with the bell-shaped “swelling vs. Q” plots in Figure 4 (gray and black columns).

## 4. Conclusions

Six hydrophilic cross-linked copolymers were synthesized with the same content of carboxyl groups and the content of cross-linking agent Q varied from 0.04 up to 1 wt.%. The Q value is of key parameter, which determines the behavior of copolymers at their swelling in water and turning them into hydrogels. In the aqueous-salt solution, an increase in Q accelerates the swelling but decreases an equilibrium degree of swelling. The situation changes drastically when the copolymers are mixed with a solid substrate, sand or soil, and then water is added. This results in additional acceleration of swelling whereas equilibrium degree of swelling shows the maximum for the copolymer with Q = 0.14 wt.%. Rheological study indicates the viscoelastic behavior of all hydrogels with different Q values. The storage modulus G′, related to the mechanical behavior the hydrogels, shows a linear rise up to Q = 0.3 wt.% and then increases only slightly. The swelling pressure of hydrogels and water-retaining properties of solid substrates with embedded hydrogels pass through a maximum at Q = 0.14 wt.%. Addition of the hydrogels to the sand and soil significantly improves the hydrophysical parameters of solid substrates, which are responsible for plant germination and development.

We see therefore a pronounced relationship between the degree of copolymer cross-linking, on the one hand, and the key characteristics of copolymer hydrogels in the solid substrate—equilibrium degree of swelling, the maximum pressure in the polymer network and water-retaining properties, on the other hand. These characteristics acquire their maximum values at Q = 0.14 wt.% when a balance is reached between the elasticity of swollen polymer gels and the resistance from solid particles. These previously undescribed dependences set the algorithm for synthesis of polymeric superabsorbents and optimization of their operational characteristics.

## Figures and Tables

**Table 1:** Water-retaining properties of sand and soil with 1 wt.% content of ACP#s. The samples were saturated with 10^−3^ M phosphate buffer, pH 6.5.

Hydrophysical Indicator	Q, wt.%						
	0	0.04	0.08	0.14	0.2	0.4	1
**SAND**							
**W_max_**	27	91	104	136	116	99	83
**FWC**	4	52	46	61	60	54	47
**WP**	1	30	23	30	34	31	29
**AWR = FWC − WP**	3	21	23	31	26	23	18
**SOIL**							
**W_max_**	36	79	84	127	98	84	73
**FWC**	14	41	45	65	55	47	44
**WP**	5	14	18	32	27	23	22
**AWR = FWC − WP**	9	27	27	33	28	24	22

## Data Availability

The data presented in this study are available on request from the corresponding author. The data are not publicly available due to privacy.
